# Noninvasive temporal detection of early retinal vascular changes during diabetes

**DOI:** 10.1038/s41598-020-73486-2

**Published:** 2020-10-15

**Authors:** Mohammad Ali Saghiri, Andrew Suscha, Shoujian Wang, Ali Mohammad Saghiri, Christine M. Sorenson, Nader Sheibani

**Affiliations:** 1 Director of Biomaterial and Prosthodontic Laboratory, Department of Restorative Dentistry, Rutgers School of Dental Medicine, Rutgers Biomedical and Health Sciences, MSB C639A, 185 South Orange Avenue, Newark, NJ 07103 USA; 2 Department of Endodontics, University of the Pacific, Arthur A. Dugoni School of Dentistry, San Francisco, CA USA; 3 Department of Ophthalmology and Visual Sciences, School of Medicine and Public Health, University of Wisconsin, Madison, WI USA; 4 Tehran, Iran; 5 Department of Pediatrics, School of Medicine and Public Health, University of Wisconsin, Madison, WI USA; 6 Department of Cell and Regenerative Biology, School of Medicine and Public Health, University of Wisconsin, Madison, WI USA; 7 Department of Biomedical Engineering, School of Medicine and Public Health, University of Wisconsin, Madison, WI USA

**Keywords:** Oncology, Diagnosis, Disease prevention, Occupational health

## Abstract

Diabetes associated complications, including diabetic retinopathy and loss of vision, are major health concerns. Detecting early retinal vascular changes during diabetes is not well documented, and only few studies have addressed this domain. The purpose of this study was to noninvasively evaluate temporal changes in retinal vasculature at very early stages of diabetes using fundus images from preclinical models of diabetes. Non-diabetic and Akita/+ male mice with different duration of diabetes were subjected to fundus imaging using a Micron III imaging system. The images were obtained from 4 weeks- (onset of diabetes), 8 weeks-, 16 weeks-, and 24 weeks-old male Akita/+ and non-diabetic mice. In total 104 fundus images were subjected to analysis for various feature extractions. A combination of Canny Edge Detector and Angiogenesis Analyzer plug-ins in ImageJ were utilized to quantify various retinal vascular changes in fundus images. Statistical analyses were conducted to determine significant differences in the various extracted features from fundus images of diabetic and non-diabetic animals. Our novel image analysis method led to extraction of over 20 features. These results indicated that some of these features were significantly changed with a short duration of diabetes, and others remained the same but changed after longer duration of diabetes. These patterns likely distinguish acute (protective) and chronic (damaging) associated changes with diabetes. We show that with a combination of various plugging one can extract over 20 features from retinal vasculature fundus images. These features change during diabetes, thus allowing the quantification of quality of retinal vascular architecture as biomarkers for disease progression. In addition, our method was able to identify unique differences among diabetic mice with different duration of diabetes. The ability to noninvasively detect temporal retinal vascular changes during diabetes could lead to identification of specific markers important in the development and progression of diabetes mediated-microvascular changes, evaluation of therapeutic interventions, and eventual reversal of these changes in order to stop or delay disease progression.

## Introduction

Many studies have now demonstrated that the eyes provide a window for assessing the health and integrity of the systemic vasculature and the central nervous system, as several diseases present signature changes in the retinal vasculature features. Nowhere else in the body can internal health be noted without substantial intervention. During manual ocular examination, some features like the health of optic disc, nerves and macula, and retinal blood vessel architecture can be easily detected. Patients are commonly examined for changes in the retinal arteries and nerves by shining a light through the iris on to the back of the eye using an ophthalmoscope. Traditionally, manual observations via an ophthalmoscope has been utilized for various diagnoses^1^. However, recently new hardware and software are used to digitalize ocular fundus images^2^. This digitalization of images is allowing for more precise measurements of ocular changes, creating opportunities to identify and monitor ocular and systemic diseases earlier than traditional methods^3^. Building on this trend towards digitalized retinal vascular analysis, here we investigated noninvasive fundus imaging to quantify changes in the retinal vasculature architecture during diabetes.

The retina relies on a well-functioning and adequate blood supply for its proper function without interruption or incident. However, many diseases interfere with the body’s ability to supply retinal cells with blood through disruption of microvascular integrity and function. In the case of cardiovascular disease (CVD), it is known that the initial disease progression often begins with vascular cell dysfunction, which leads to eventual inflammation-induced disruption of the microvasculature integrity^4^. This damage to the blood vessels is also associated with several physiological pathways such as elevated blood pressure, diabetes, inflammation, and raised blood lipids. These abnormalities eventually progress to atherosclerosis and CVD, making early diagnosis imperative for clinical treatment^5^.

Due to the known alterations in microvascular integrity as a common precursor to CVD, many investigations have begun using the eye as a window into the body’s systemic stress. In a systematic review of such studies Li et al.^6^ found evidence that vascular structural changes were associated with CVD, which were reflected through retinal vasculature analysis^6^. Similarly, a systematic review by Kee et al.^7^ found an association between reduced retinal microvasculature integrity and type 1 diabetes severity, a risk factor for CVD in humans^7^. Together, these findings support the potential use of digitalized retinal vasculature analysis for early diagnosis of various vascular dysfunction and potential systemic diseases such as diabetes.

Diabetes Mellitus (DM) is a prevalent systemic disease, with 34.2 million people affected in the US alone and is considered to be the 7th leading cause of death^8^. Diabetes affects different systems of the body to varying degrees, especially the cardiovascular system^9^. One of the other complications of DM is diabetic retinopathy (DR), which is one of the leading causes of blindness in adults^10^. According to published studies, an estimated 4.1 million people have DR that is 1 in every 29 person^11^. DR presents itself in the form of retinal ischemia, including intra-retinal microvascular abnormalities and neovascularization^12^. However, how these changes are brought about and whether detection of changes in vascular features early during the disease can be used to predict such later complications remain unknown.

The potential to utilize digitalized ocular image analysis in early diagnosis relies on changes in retinal vascular organization, which are detectable by various analysis. In DR, for instance early oxidative stress and inflammation leads to damaged endothelium and eventually capillary degeneration and ischemia^13^. While later edema stages of DR are easily diagnosed using a traditional ophthalmoscope as they later present themselves as proliferative DR^14^. The early changes in retinal metabolic activity lead to microvascular alterations (non-proliferative abnormalities), which follow similar inflammatory pathways associated with DM progression^15–17^. These features can be detected using various methods both physically as well as digitally, where one study outlines an automated method to detect these abnormalities and classify them^18^. Due to changes in microvascular integrity in early DR, as well as the known ability to track CVD and DR using retinal vascular changes, we believe that digitalized retinal fundus image analysis has the potential for earlier diagnosis of diabetes-mediated vascular changes.

Several programs, which analyze microvasculature structures already exist as macros in ImageJ. One such program, the Angiogenesis Analyzer, was developed to analyze the three- dimensional network organization of endothelial cells in culture^19^. A particularly useful feature of this plugin is its ability to analyze binary images of microvasculature. This binary analysis allows for consistent quantification of microvascular density and the overall interconnectedness of blood vessels. Moreover, when the Angiogenesis Analyzer is used in conjunction with Canny Edge Detector^20^, an ImageJ plugin which can extract microvasculature features in binary format, one can consistently extract previously unquantifiable features of microvasculature.

Through the novel application of the Angiogenesis Analyzer to retinal microvasculature following Canny Edge Detection, we aimed to identify features that can aid in quantification of the early signature changes in retinal vasculature during diabetes. We believe that a combination of these aforementioned programs can have a significant impact on preserving vision and treatment of visual complications due to their ability to detect minute microvasculature alterations. This notion is further supported by the known ability to detect retinal microvascular changes associated with diabetes. Thus, we proposed our combination of vessel analysis program that could be used to quantify early microvascular changes in mice with DM. Here we used a combination of vessel analysis methods available through the NIH to quantify the retinal vasculature architecture. To help better recognize the vascular-based abnormalities, we provide guided steps on how to quantify the edge of vessels and extract over 20 features of retinal vasculature architecture.

## Material and methods

### Mice

Wild type (C57BL/6J) and heterozygous (Ins2^Akita/+^_;_ C57BL/6J background) mice were obtained from Jackson Laboratory (Bar Harbor, ME). Only the male mice that are heterozygous for Akita mutation (Akita/+) develop diabetes and were used in our studies. In order to breed more of the Ins2^Akita/+^ mice, C57BL/6J inbred females were bred with Ins2^Akita/+^ males to generate the animals needed for the study. Control animals were C57BL/6J male mice. Control animals are similar (wild type; + /+) mice without hyperglycemia. This model is used in many previous studies^21–23^ to investigate the pathophysiology of diabetic retinopathy. The animals have unlimited access to food and water. Commonly animals with glucose levels greater than 250 mg/dl are considered diabetic, while non-diabetic controls have glucose levels of less than/or 110 mg/dL^21–23^. The Akita/+ mice become diabetic at 4 weeks of age with a blood glucose level of > 420 mg/dl. The majority of early eye retinopathies in these mice occur after 6 months of diabetes^13^. Genomic DNA was prepared from tail biopsies and the transgenic Akita/+ mice were identified by PCR screening as previously described^24^.

Here we used the Akita/+ mice, which develop diabetes spontaneously, and is a well-accepted diabetes model for investigating the pathophysiology of diabetic retinopathy^24–27^. The STZ model is also a valid model and is used extensively for similar evaluations^28–32^. The systemic effect of STZ is mainly on the beta-cells of the pancreas and not necessarily on the retinal vasculature because the diabetes mediated vascular changes take months to present. STZ is chemically very unstable and is rapidly inactivated, thus it will not stay around to cause damage. Similar vascular changes of later stages are noted in the majority of rodent diabetes models. In addition, the Akita/+ model has the advantage of not using STZ and avoiding its potential adverse systemic effects. Thus, the vascular changes seen here are mediated by the effects of diabetes and are not because of STZ use. All animal procedures were performed in accordance with the Association for Research in Vision and Ophthalmology statement for the use of animals in vision research and approved by the Institutional Animal Care and Use Committee of the University of Wisconsin School of Medicine and Public Health.

### Study design

This study follows the experimental research design. In this study, the mice were divided into two groups, diabetic (Akita/+ mice; n = 9) and the control group (C57BL/6J male mice; n = 9). The in vivo fundus imaging was performed on both the left and right eyes of both groups of mice using the Micron III retinal imaging system (Phoenix Laboratories, Inc., Pleasanton, CA) as recommended by the supplier. Fundus images were taken for each mouse at 4 weeks, 8 weeks, 16 weeks, and 24 weeks of age. The independent variable in this study is diabetes condition in the mice, and the dependent variables are the various features chosen for this study, which will be observed from the fundus images. Both groups of mice were prepared using the same procedure as described and were imaged and analyzed similarly as well as a control for the experimental procedure. To ensure equal depth of focus for each image, all fundus images were taken with the same magnification, such that the circular border of the image is distinctly focused. Fundus images were taken for each mouse at 4 weeks, 8 weeks, 16 weeks, and 24 weeks of age. These time points were selected for modeling diabetes in earlier stages, and are based on our previous studies and those used by other investigators in published studies.

### Fundus imaging and analysis

**Figure 1:**
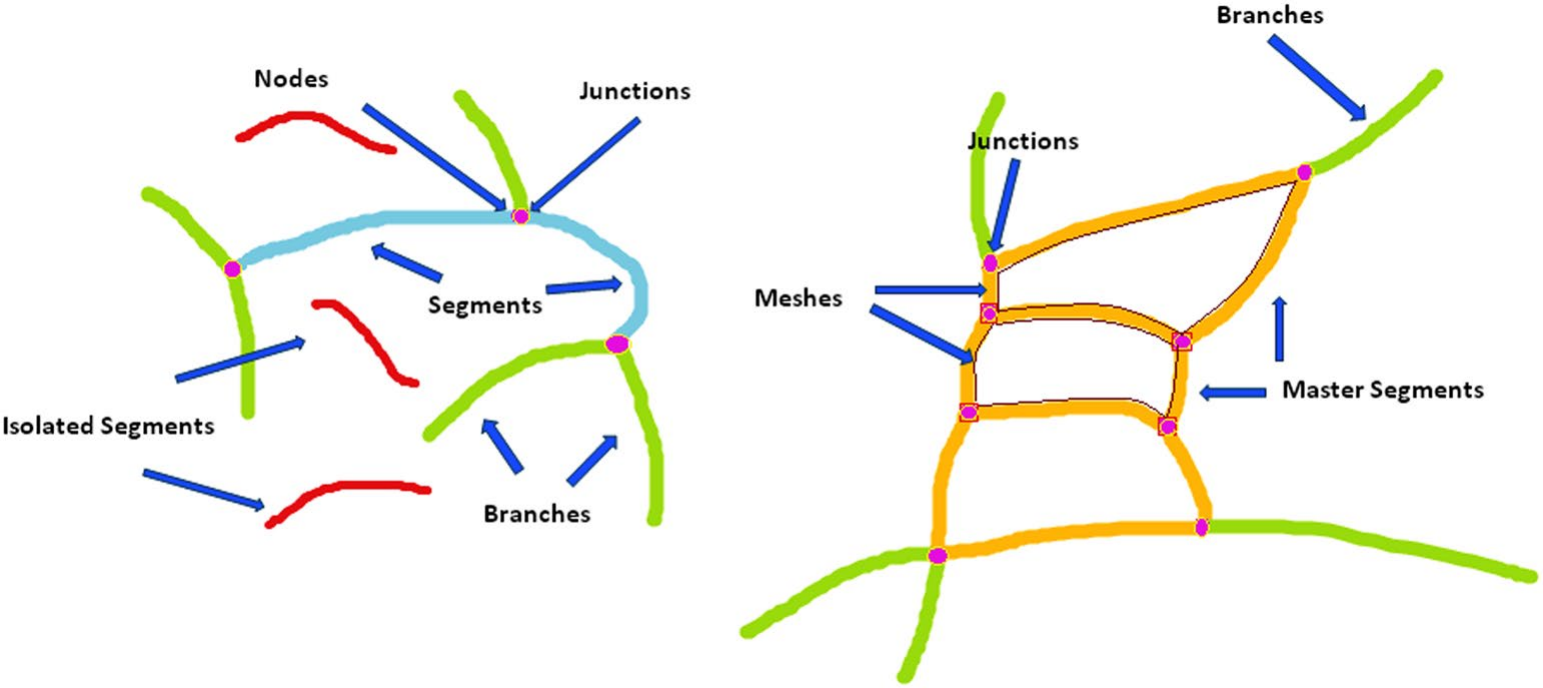
Cartoon representation of features extracted from Angiogenesis Analyzer**.** This cartoon representation depicts the features extracted by the Angiogenesis Analyzer program. Fundus analysis metrics are further defined in Table 1.

In preparation for the imaging process, briefly, the mice were weighed and anesthetized with IP injection of ketamine (100 mg/kg) and xylazine (10 mg/kg), and their pupils were dilated using 1% tropicamide (Bausch and Lomb, Inc., Tampa, FL). Mice were kept warm on a heating pad during imaging. Once these images were obtained, they were analyzed similarly to the studies performed as previously described by Saghiri et al.^33^, and used the "Angiogenesis Analyzer" plug-in for Angiogenesis Analyzer for ImageJ^34,35^, which allows analysis of cellular networks. These programs can detect and analyze the pseudo vascular organization, creating an output of features defined in Table 1 and depicted in Fig. 1. Detailed software description and guided steps are provided in the supplemental material.

**Table 1:** Definition of features extracted from Angiogenesis Analyzer.

Feature	Definition*
Extremities	Entire interconnected network of vasculature, all branches in the analyzed tree
Nodes	Pixel with at least 3 neighbors
Junctions	Multiple nodes together
Branches	Extremity connected to other vasculature with only a single junction
Segments	Extremities connected by two junctions
Isolated Segments	Elements that are not connected to the main tree
Master Segments	Tree segments that are exclusively connected to branches and other master segments
Master Junctions	Junctions connecting at least three master segments
Meshes	Area in between master junctions
Mesh Index	Average distance between two master junctions (Tot. master segments length/Nb master segments)
Mean Mesh Size	Average of all mesh sizes being analyzed
Branching Interval	Mean distance separating two branches in a tree (Tot. segments length/Nb branches)
Pieces	Combination of number of segments, isolated segments, and branches detected in the analyzed area
Branching Length	Combination of length of the trees composed from segments and branches in the analyzed area

*Description of features extricated from fundus image analysis and depicted in Fig. 1. Several features were quantified by both their total number and their total length.

### Preparation of STZ mediated diabetic mice

Thrombospondin-1 deficient (TSP1−/−) mice exhibit an inherent sensitivity to diabetes mediated retinal vascular changes with a shorter duration of diabetes^24^. TSP1−/− mice were generated and maintained as previously described^36^. Eight week-old male TSP1−/− were made diabetic by a single injection of STZ prepared fresh in citrate buffer (150 mg/kg). Control (non-diabetic) animals received citrate buffer alone. The blood sugars were measured using a glucometer (Glucometer Elite, Bayer Inc., Toronto, Ont.) and animals with glucose levels ≥ 250 mg/dL were considered diabetic^13,24,37^. A total of 8 diabetic and 7 non-diabetic mice were used for these analysis. Fundus images were captured using a Micron III imaging system as described above before STZ treatment, and after 12 weeks of diabetes. Following fundus imaging after 12 weeks of diabetes, mice were sacrificed and retinas were harvested for whole-mount trypsin digest preparation and quantification of endothelial cells, pericytes density and their ratio as previously described^24,38,39^.

### Statistical analysis

A Student’s unpaired t-test (two-tailed) compared statistical differences between diabetic (n = 9) and control (n = 9) mice at each time point using GraphPad Prism version 5.04 for Windows (GraphPad Software, La Jolla, CA). P < 0.05 is considered significant. Mean ± standard deviation is reported. The sample size was calculated using a free open source software called G Power. In this software, the appropriate test was selected (t-test—family, Means: Difference between two independent means—Statistical test). The number of tails were set as 2, with the effect size 1.5, Type 1 error as 0.05, power as 0.8 and allocation ratio was set as 1. The sample size for both groups 1 and 2 were calculated by the software to be set as 9 each, with the total sample size being 18. The actual power calculated was 0.8476.

## Results

### Image analysis

Fundus images were obtained from non-diabetic and diabetic Akita/+ mice at indicated time points from same group of animals. Fundus images were then subjected to analysis for extraction and quantification of changes in various vascular parameters (Supplementary Data and Fig. 1, Table 1). The changes in these parameters were compared between non-diabetic and diabetic mice at indicated time points. The significance of these changes during diabetes was determined by statistical comparison of our findings in fundus images from diabetic compared with non-diabetic mice, and are summarized below.

### Mesh number, area, mean size, and index

**Figure 2:**
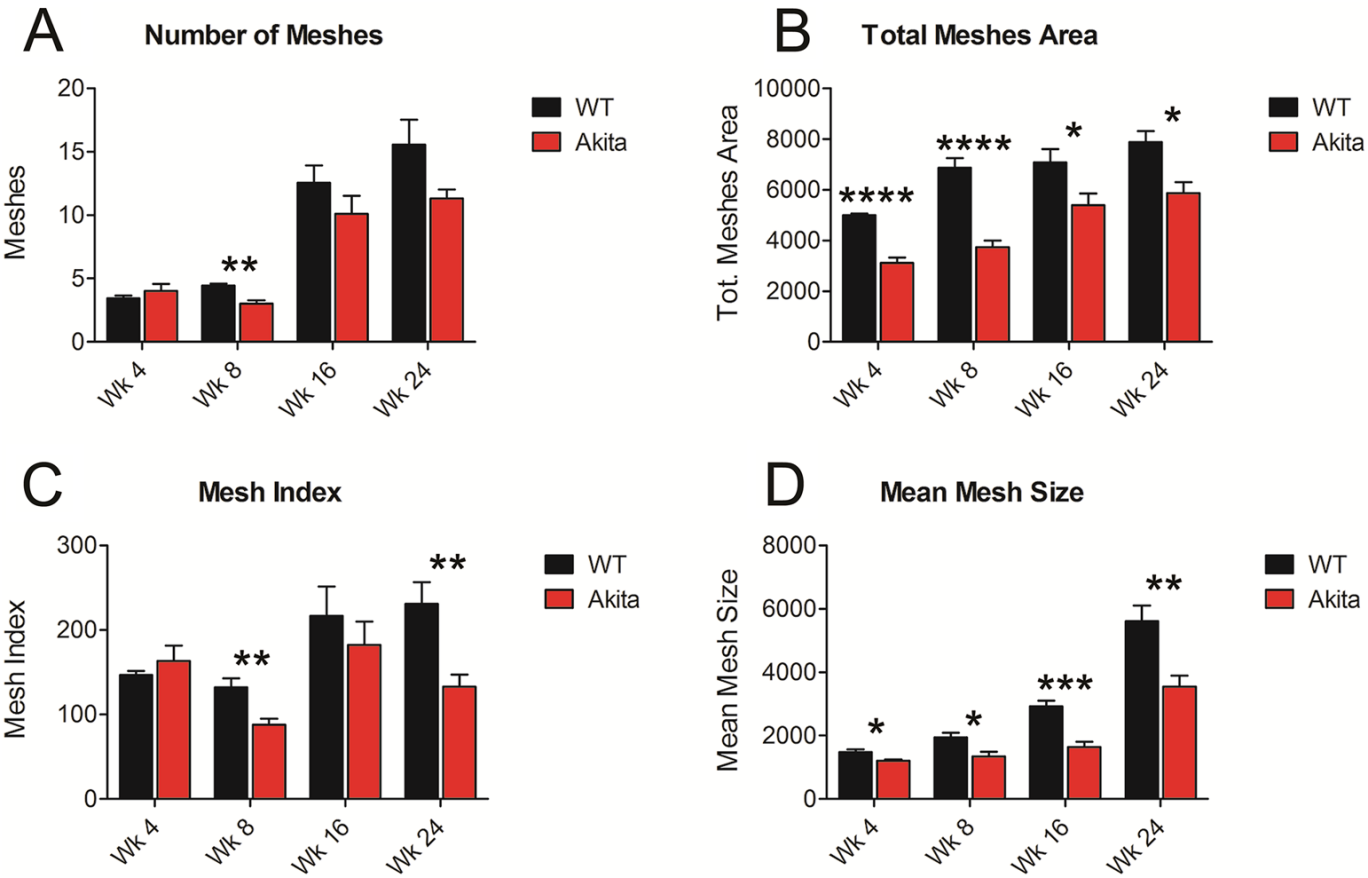
Mesh Measurements. The following metrics were determined and compared in fundus images from diabetic and non-diabetic mice using our image analysis. (**A**) Number of Meshes at week 0—WT: 3.44 ± 0.7265, Akita/+ : 4 ± 1.936 (P = 0.4322); week 8—WT: 4.444 ± 0.527, Akita/+ : 3.00 ± 1.00 (P = 0.0015); week 16—WT: 12.56 ± 4.558, Akita/+ 10.11 ± 4.755 (P = 0.282); and week 24—WT: 15.56 ± 6.616, Akita/+ 11.33 ± 2.345 (P = 0.09). (**B**) Total mesh area at week 0—WT: 5007 ± 207.8, Akita/+ : 3115 ± 706 (P < 0.0001); week 8—WT: 6874 ± 1258, Akita/+ : 3737 ± 870.9 (P < 0.0001); week 16—WT: 7078 ± 1766, Akita/+ : 5394 ± 1550 (P = 0.0473); and week 24—WT: 7883 ± 1472, Akita/+ : 5862 ± 1471 (P = 0.0101). (**C**) Mesh index at week 0—WT: 147 ± 15.04, Akita/+ : 163.3 ± 60.42 (P = 0.4426); week 8—WT: 132 ± 36.25, Akita/+ : 87.98 ± 23.32 (P = 0.0074); week 16—WT: 217 ± 115, Akita/+ : 182.5 ± 91.7 (P = 0.4917); and week 24—WT: 230.9 ± 86.15, Akita/+ : 132.8 ± 49.41 (P = 0.0079). (**D**) Mean mesh size at week 0—WT: 1484 ± 263.6, Akita/+ : 1205 ± 148 (P = 0.0139); week 8—WT: 1948 ± 482.7, Akita/+ : 1349 ± 487.7 (P = 0.0187); week 16—WT: 2925 ± 593.5, Akita/+ : 1635 ± 572.6 (P = 0.0002); and week 24—WT: 5607 ± 1659, Akita/+ 3544 ± 1190 (P = 0.0079). Initially, the differences in the total mesh area and mean mesh size were statistically significant. At week 8, the differences in all mesh metrics were statistically significant. At week 16, the differences in total mesh area and mean mesh size were statistically significant. At week 24, the differences in total mesh area, mesh index, and mean mesh size were statistically significant. WT: Wild type non-diabetic.

The total mesh number, an extracted feature where connected vasculature surrounds a vascular fundus area on all sides, was greater in non-diabetic mice compared with Akita/+ mice at weeks 8, 16, and 24 (Fig. 2A). However, the differences were only statistically significant at week 8 (P = 0.0015). The total mesh area or total surface area, which is surrounded by the vasculature on all sides, was statistically greater in non-diabetic compared with Akita/+ mice at each time point; initially (P < 0.0001), week 8 (P < 0.0001), week 16 (P = 0.0473), and week 24 (P = 0.0101) (Fig. 2B). Non-diabetic animals had a statistically greater mean mesh size, which represents the average size of a single mesh feature; initially (P = 0.0139), week 8 (P = 0.0187), week 16 (P = 0.0002), and week 24 (P = 0.0079) (Fig. 2C). Following a different trend, mesh index, determined by the total master segment length over the number of master segments, displayed a significant difference at week 8 (P = 0.0074) and week 24 (P = 0.0091). No significant differences were noted initially or at week 16 (Fig. 2D).

### Extremities and nodes

**Figure 3:**
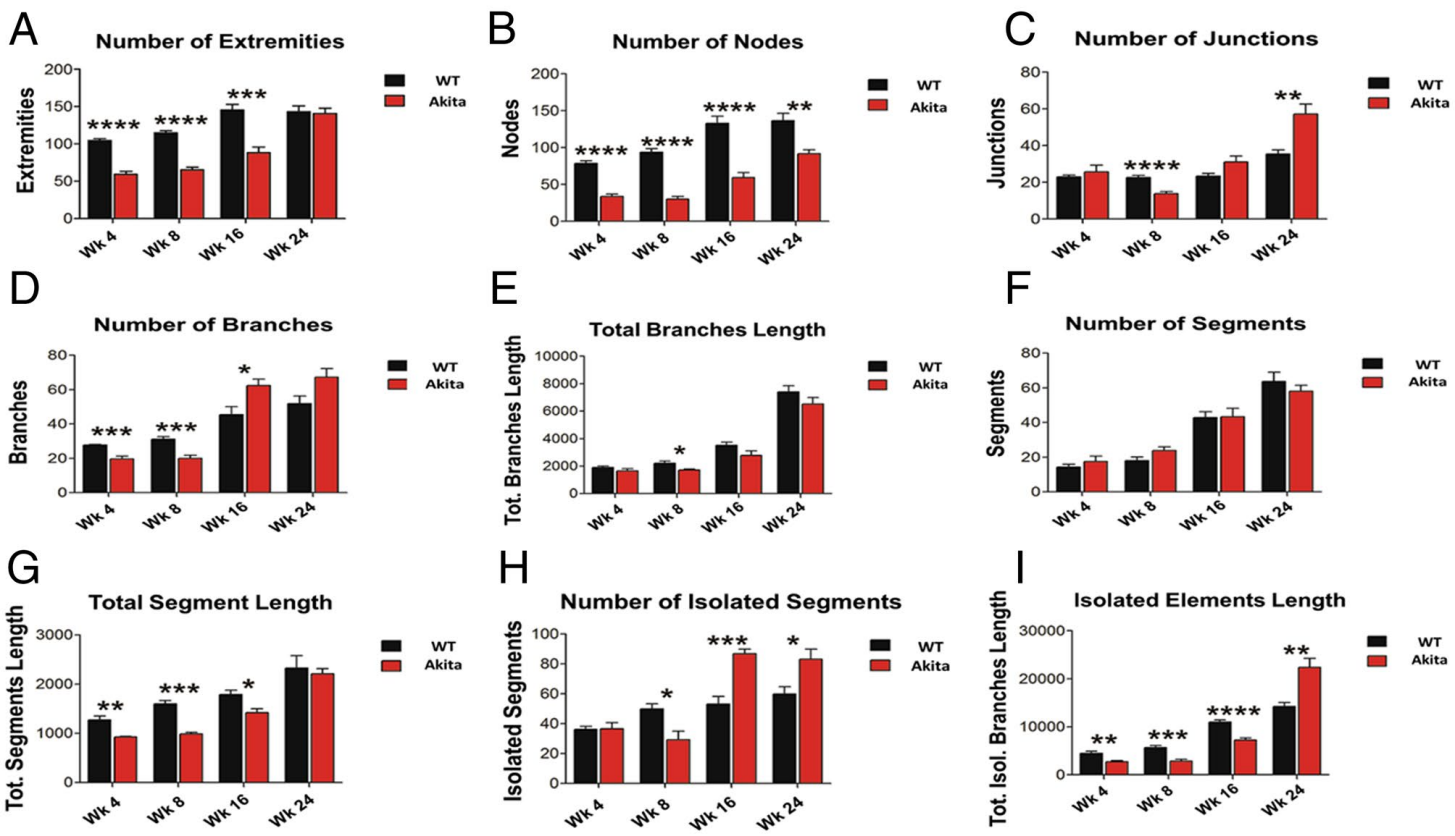
Measurements of other parameters. The following metrics were determined and compared in fundus images from diabetic and non-diabetic mice using our image analysis. (**A**) Number of Extremities at week 0—WT: 104.2 ± 8.348, Akita/+ : 59 ± 12.93 (P < 0.0001); week 9—WT: 114.7 ± 9.11, Akita/+ : 64.89 ± 12.62 (P < 0.0001); week 16—WT: 145.2 ± 25.95, Akita/+ : 88.11 ± 24.87 (P = 0.0002); and at week 24—WT: 143 ± 27.04, Akita/+ : 140.7 ± 22.95 (P = 0.846). (**B**) Number of Nodes at week 0—WT: 78.44 ± 12.17, Akita/+ 33.44 ± 11.16 (P < 0.0001); week 8—WT: 93.33 ± 17.74, Akita/+ : 30 ± 12.29 (P < 0.0001); week 16—WT: 132.7 ± 33.4, Akita/+ : 59.22 ± 23.17 (P < 0.0001); and week 24—WT: 136 ± 35.02, Akita/+ : 91.56 ± 17.5 (P = 0.0035). (**C**) Number of Junctions at week 0—WT: 22.89 ± 2.892, Akita/+ : 25.56 ± 11.11 (P = 0.0496); week 8—WT: 22.56 ± 3.644, Akita/+ : 13.67 ± 3.606 (P < 0.0001); week 16—WT: 23.33 ± 4.77, Akita/+ : 31 ± 9.912 (P = 0.0528); and week 24—WT: 35.33 ± 7.583, Akita/+ : 57.11 ± 16.14 (P = 0.0021). (**D**) Number of Branches at week 0—WT: 27.78 ± 1.302, Akita/+ : 19.78 ± 5.357 (P = 0.0005); week 8—WT: 31.33 ± 4.416, Akita/+ : 20 ± 6.205 (P = 0.0004); week 16—WT: 45.33 ± 15.87, Akita/+ : 62.44 ± 12.24, P = 0.0209; and week 24—WT: 51.89 ± 14.47, Akita/+ : 67.22 ± 16.73 (P = 0.054). (**E**) Total Branch Length at week 0—WT: 1905 ± 302.5, Akita/+ : 1653 ± 543.5 (P = 0.2416); week 8—WT: 2203 ± 554.6, Akita/+ : 1701 ± 299.3 (P = 0.0296); week 16—WT: 3521 ± 722.7, Akita/+ 2784 ± 1076 (P = 0.1073); and week 24—WT: 7390 ± 1506, Akita/+ : 6500 ± 1565 (P = 0.2369). (**F**) Number of Segments at week 0—WT: 14.33 ± 5.809, Akita/+ : 17.56 ± 3.432 (P = 0.4256); week 8—WT: 18.11 ± 7.132, Akita/+ : 23.89 ± 2.406 (P = 0.1069); week 16—WT: 42.78 ± 11.36, Akita/+ : 43.33 ± 5.302 (P = 0.9331); and week 24—WT: 63.67 ± 18.06, Akita/+ : 58.00 ± 3.775 (P = 0.4369). (**G**) Total Segment Length at week 0—WT: 1271 ± 280.1, Akita/+ : 925.3 ± 51.22 (P = 0.0022); week 8—WT: 1600 ± 235.2, Akita/+ : 992.3 ± 96.78 (P < 0.0001); week 16—WT: 1792 ± 291.7, Akita/+ : 1421 ± 278.1 (P = 0.0138); and week 24—WT: 2326 ± 857.1, Akita/+ : 2209 ± 367.3 (P = 0.7102). (**H**) Number of Isolated Segments at week 0—WT: 36.11 ± 6.936, Akita/+ : 17.56 ± 10.3 (P = 0.93); week 8—WT: 49.89 ± 11.27, Akita/+ : 29.11 ± 6.52 (P = 0.0139); week 16—WT: 53 ± 17.2, Akita/+ : 86.89 ± 9.905 (P = 0.0001); and week 24—WT: 59.78 ± 16.77, Akita/+ : 83.11 ± 22.78 (P = 0.0249). (**I**) Isolated Element Length at week 0—WT: 448 ± 1533, Akita/+ : 2739 ± 837.5 (P = 0.0084); week 8—WT: 5645 ± 1580, Akita/+ : 2835 ± 1264 (P = 0.0007); week 16—WT: 10,958 ± 1554, Akita/+ : 7224 ± 1505 (P < 0.0001); and week 24—WT: 14,234 ± 2725, Akita/+ : 22,362 ± 6383 (P = 0.0029). Initially, the differences in number of extremities, number of nodes, number of branches, total segments length, and isolated elements length were statistically significant. At week 8, the differences in number of extremities, number of nodes, number of junctions, number of branches, total branches length, number of isolated segments, total segments length, and isolated elements length were statistically significant. At week 16, the differences in number of extremities, number of nodes, number of branches, number of isolated segments, total segments length, and isolated elements length were statistically significant. At week 24, the differences in number of nodes, number of junctions, number of isolated segments, and isolated elements length were statistically significant.

Total number of extremities for non-diabetic mice was significantly higher compared with Akita/+ mice initially (P < 0.0001), at week 8 (P < 0.0001), and at week 16 (P = 0.0002). No significant difference was found for total number of extremities at week 24 (Fig. 3A). The non-diabetic mice demonstrated a significantly greater total number of nodes initially (P < 0.0001), at week 8 (P < 0.0001), week 16 (P < 0.0001), and week 24 (P ≤ 0.0035) (Fig. 3B).

### Junctions

The total number of junctions initially showed minimal differences between non-diabetic and Akita/+ mice (Fig. 3C). Non-diabetic mice displayed a significantly greater total number of junctions than Akita/+ mice at week 8 (P < 0.0001). Akita/+ mice had greater total number of junctions than non-diabetic mice at 16 weeks, but this difference was not statistically significant (P = 0.0528). At week 24, both non-diabetic and Akita/+ mice showed greater total number of junctions than their previous measurements. However, Akita/+ mice demonstrated a greater difference between the two time points. Akita/+ mice had significantly greater total number of junctions than non-diabetic mice by week 24 (P = 0.0021).

### Branches

Non-diabetic mice had a significantly larger total number of branches initially (P = 0.0005) and at week 8 (P = 0.0004) (Fig. 3D). These mice showed little change in total number of branches between week 4 and week 8. Both non-diabetic and Akita/+ mice showed increased total number of branches in week 16 compared with week 8. However, Akita/+ mice showed an increase by a much greater margin than non-diabetic mice. Akita/+ mice had significantly greater total number of branches compared to non-diabetic mice at week 16 (P = 0.0209). Akita/+ mice maintained greater but not statistically significant total number of branches than non-diabetic mice in week 24 (P = 0.054). Neither non-diabetic nor Akita/+ mice showed significant changes in total number of branches between week 16 and week 24.

The total length of all branches initially showed no significant differences between non-diabetic and Akita/+ mice. Both groups increased total branch length in week 8 compared to their initial measurement. Non-diabetic mice showed an increase with a larger margin than Akita/+ mice, leading to non-diabetic mice having significantly greater total branch length than Akita/+ mice in week 8 (P = 0.0296). Both non-diabetic and Akita/+ mice showed increased total branch length by similar amounts in week 16 and week 24 (Fig. 3E). While non-diabetic mice maintained their greater total branch length at these time points, no statistically significant differences were noted.

### Segments

No significant differences in the total number of segments were noted between non-diabetic and Akita/+ mice at any of the time points evaluated (Fig. 3F). Non-diabetic mice had a greater total length of segments than Akita/+ mice: initially (P = 0.0022), at week 8 (P < 0.0001), and at week 16 (P = 0.0138). Both non-diabetic and Akita/+ mice showed a modest increase at each time point. No significant differences were observed between non-diabetic and Akita/+ mice for total segment length at week 24 (Fig. 3G).

### Isolated segments

No significant differences were initially observed between non-diabetic and Akita/+ mice for total number of isolated segments, defined as segments which are not connected to any larger vascular network. Week 8 measurements showed an increase for non-diabetic and a decrease for Akita/+ mice in total number of isolated segments compared with week 4. Non-diabetic mice had greater total number of isolated segments than Akita/+ mice in week 8 (P = 0.0139). Both non-diabetic and Akita/+ mice showed increased total number of isolated segments in week 16 compared with week 8. However, Akita/+ mice showed an increase by a much larger margin. Akita/+ mice had greater total number of isolated segments than non-diabetic mice in week 16 (P = 0.0001). Non-diabetic and Akita/+ mice showed minimal changes in total number of isolated segments from week 16 to week 24. The total number of isolated segments for Akita/+ mice remained greater than non-diabetic mice in week 24 (P = 0.0249) (Fig. 3H).

The changes in the total isolated element length; total length of all isolated segments, were also recorded where it was observed that non-diabetic mice initially had significantly greater total isolated element length, than Akita/+ mice (P = 0.0084). Minimal changes were observed for total isolated element length in week 8 for both Akita/+ and non-diabetic mice. The non-diabetic mice total isolated element length remained greater than Akita/+ mice in week 8 (P = 0.0007). Both non-diabetic and Akita/+ mice showed increased total isolated element length in week 16 compared with week 8 at similar rates. The total isolated element length in non-diabetic mice remained greater than Akita/+ mice in week 16 (P < 0.0001). Week 24 measurements showed both non-diabetic and Akita/+ mice had increased total isolated element length compared with week 16. However, Akita/+ mice showed an increase at a much greater margin. Akita/+ mice had greater total isolated branch length than non-diabetic mice in week 24 (P = 0.0029) (Fig. 3I).

### Pieces

**Figure 4:**
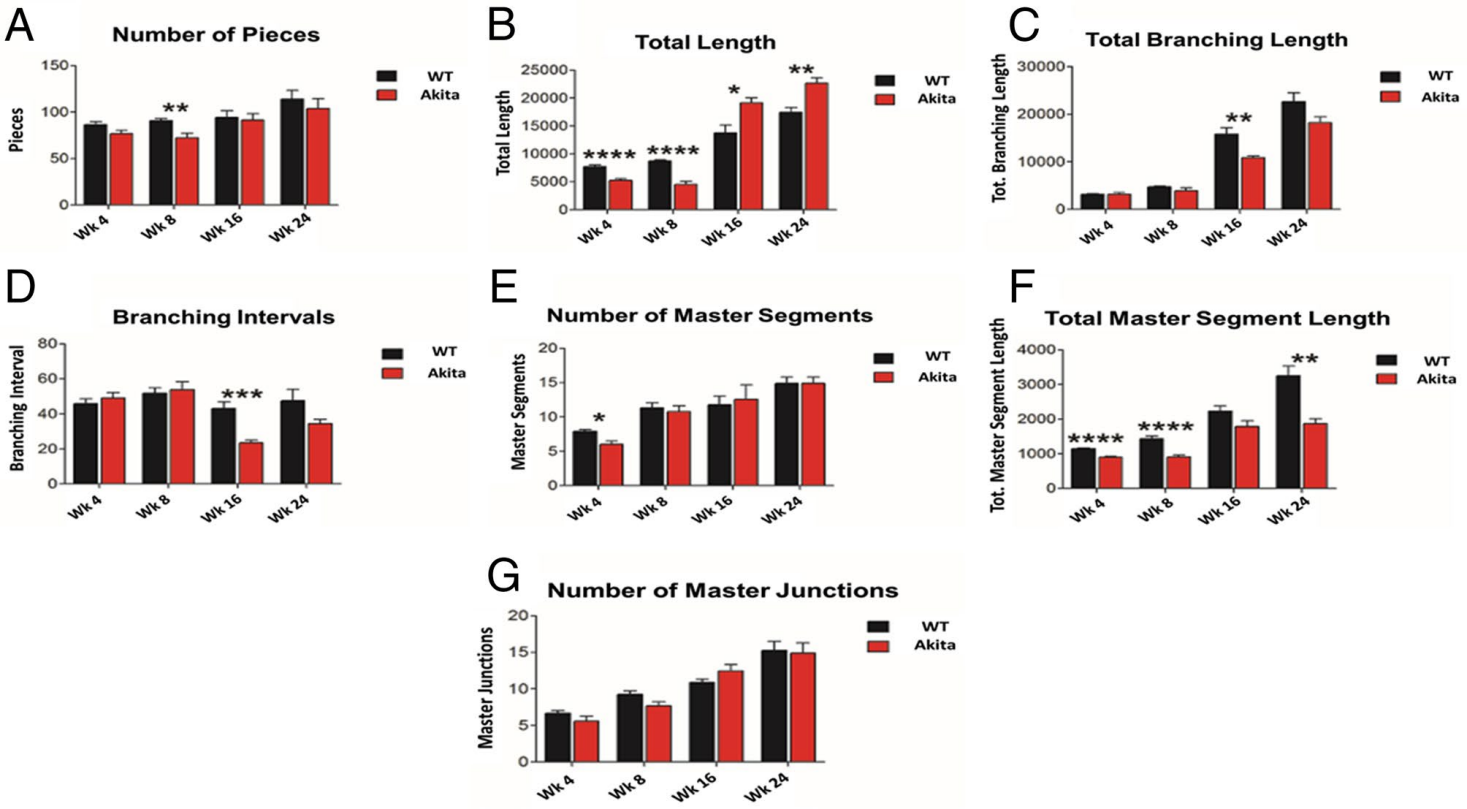
Measurements of additional parameters. The following metrics were determined and compared in fundus images from diabetic and non-diabetic mice using our image analysis. (**A**) Number of Pieces at week 0—WT: 86.44 ± 10.33, Akita/+ : 76.78 ± 12.37 (P = 0.0908); week 8—WT: 90.78 ± 7.12, Akita/+ : 72.33 ± 15.75 (P = 0.0056); week 16—WT: 94.22 ± 25.16, Akita/+ : 91.33 ± 26.61 (P = 0.8049); and week 24—WT: 114 ± 31.16, Akita/+ : 103.8 ± 35.92 (P = 0.5281). (**B**) Total Length at week 0—WT: 7713 ± 989.9, Akita/+ : 5264 ± 922.5 (P < 0.0001); week 8—WT: 8713 ± 666, Akita/+ 4545 ± 1892 (P < 0.0001); week 16—WT: 13,754 ± 4766, Akita/+ : 19,128 ± 3160 (P = 0.0123); and week 24—WT: 17,419 ± 2862, Akita/+ : 22,654 ± 3205 (P = 0.0021). (**C**) Total Branching Length at week 0—WT: 3180 ± 373.3, Akita/+ : 3184 ± 110 (P = 0.9908); week 8—WT: 4725 ± 693.7, Akita/+ : 3908 ± 1962 (P = 0.256); week 16—WT: 15,809 ± 4450, Akita/+ : 10,835 ± 1300 (P = 0.0054); and week 24—WT: 22,594 ± 6343, Akita/+ : 18,199 ± 4032 (P = 0.0985). (**D**) Branching Interval at week 0—WT: 45.74 ± 9.49, Akita/+ : 49.04 ± 9.931 (P = 0.4815); week 8—WT: 51.84 ± 9.847, Akita/+ : 53.77 ± 15.41 (P = 0.7567); week 16—WT: 43.08 ± 12.62, Akita/+ : 23.39 ± 5.421 (P = 0.0005); and week 24—WT: 47.43 ± 22.31, Akita/+ : 34.35 ± 8.573 (P = 0.1202). (**E**) Number of Master Segments at week 0—WT: 7.889 ± 0.928, Akita/+ : 6.00 ± 0.5774 (P = 0.0108); week 8—WT: 11.33 ± 2.449, Akita/+ : 10.78 ± 2.819 (P = 0.6613); week 16—WT: 11.78 ± 4.265, Akita/+ : 12.56 ± 7.091 (P = 0.7816); and week 24—WT: 14.89 ± 3.06, Akita/+ : 15.01 ± 3.41 (P = 0.9926). (**F**) Total Master Segment Length at week 0—WT: 1149 ± 60.66, Akita/+ : 899.1 ± 88.25 (P < 0.0001); week 8—WT: 1433 ± 250.7, Akita/+ : 909 ± 161.8 (P < 0.0001); week 16—WT: 2231 ± 488.5, Akita/+ : 1790 ± 533.7 (P = 0.086); and week 24—WT: 3249 ± 942.9, Akita/+ : 1871 ± 472.9 (P = 0.0012). (**G**) Number of Master Junctions at week 0—WT: 6.667 ± 1.225, Akita/+ : 5.556 ± 2.297 (P = 0.2187); week 8—WT: 9.22 ± 1.641, Akita/+ : 7.667 ± 1.936 (P = 0.0847); week 16—WT: 10.89 ± 1.453, Akita/+ : 12.44 ± 2.92 (P = 0.1717); and week 24—WT: 15.22 ± 4.177, Akita/+ : 14.89 ± 4.649 (P = 0.8749). Initially, the differences in total length, number of master segments, and total master segment length were statistically significant. At week 8, the differences in number of pieces, total length, and total master segment length were statistically significant. At week 16, the differences in total length, total branching length, and branching interval were statistically significant. At week 24, the differences in total length, and total master segment length were statistically significant.

Pieces is a metric that represents a sum of the number segments, number of isolated elements, and number of branches. The non-diabetic mice initially had greater total number of pieces, when compared with Akita/+ mice (Fig. 4A). However, their difference was not statistically significant (P = 0.0908). The total number of pieces in non-diabetic mice increased slightly, but decreased slightly for Akita/+ mice in week 8, leading to non-diabetic mice having significantly greater total number of pieces than Akita/+ mice in week 8 (P = 0.0056). Both mice showed increased total number of pieces between weeks 8 and 16. However, Akita/+ mice had a greater margin of increase at these time points. Both Akita/+ and non-diabetic mice again showed increased total number of pieces in week 24. No statistically significant difference was found between Akita/+ and non-diabetic mice at weeks 16 or 24.

Total length, representing the total length of all pieces, was initially greater in non-diabetic mice compared with Akita/+ mice (P < 0.0001), and in week 8 (P < 0.0001). The non-diabetic and Akita/+ mice showed little changes at these time points. Week 16 non-diabetic and Akita/+ mice showed increased total length compared with week 8, however, Akita/+ mice showed an increase by a much greater margin. In week 16, Akita/+ mice had significantly greater total length than non-diabetic mice (P = 0.0123). Both non-diabetic and Akita/+ mice showed an increase in total length at week 24 compared with week 16, and Akita/+ mice showed significantly greater total length than non-diabetic mice in week 24 (P = 0.0021) (Fig. 4B).

### Vascular tree density

Total branching length represents the total length of all interconnected vasculature. It is the total length of all vasculature that is connected together in a larger “tree” of vasculature. Total branching length was similar, initially and at week 8, for non-diabetic and Akita/+ mice (Fig. 4D). By week 16, both non-diabetic and Akita/+ mice showed increased total branches length compared to week 8. The total branching length in non-diabetic mice increased by a much larger margin, leading to significantly larger total branch length (P = 0.0054) in non-diabetic than Akita/+ mice at week 16. Both non-diabetic and Akita/+ mice showed increased total branch length in week 24. The non-diabetic mice had greater total branch length compared to Akita/+ mice, however, the differences were not significant (P = 0.0985) (Fig. 4C).

Branching interval is a metric that quantifies the mean differences among branches. The branching intervals were similar, initially and at week 8 for non-diabetic and Akita/+ mice. By week 16, both non-diabetic and Akita/+ mice branching interval decreased compared with week 8. However, Akita/+ mice branching interval decreased by a much greater margin than non-diabetic mice, leading to a significant difference at week 16 (P = 0.0005). Both non-diabetic and Akita/+ mice showed modest increases in their branching interval at week 24 compared to week 16. Although non-diabetic mice maintained a greater branching interval than Akita/+ mice, no significant differences were observed (P = 0.1202) (Fig. 4D).

### Master segments and master junctions

The initial total number of master segments for non-diabetic mice was significantly higher than that of Akita/+ mice (Fig. 4F; P = 0.0108). No significant differences for total number of master segments between non-diabetic and Akita/+ mice were noted at weeks 8, 16, or 24 (Fig. 4E). The non-diabetic mice had significantly higher total master segment length compared with Akita/+ mice at week 4 (P < 0.0001), week 8 (P < 0.0001), and week 24 (P = 0.0012). Total master segment length for non-diabetic mice was also greater than that of Akita/+ mice at week 16, however, it was not statistically significant (P = 0.086) (Fig. 4F).

The non-diabetic and Akita/+ mice showed no significant differences in their initial total number of master junctions. Both groups increased total number of master junctions at week 8, week 16, and week 24 at similar rates, which were not statistically significant (Fig. 4G).

### TSP1−/− diabetic mice

**Figure 5:**
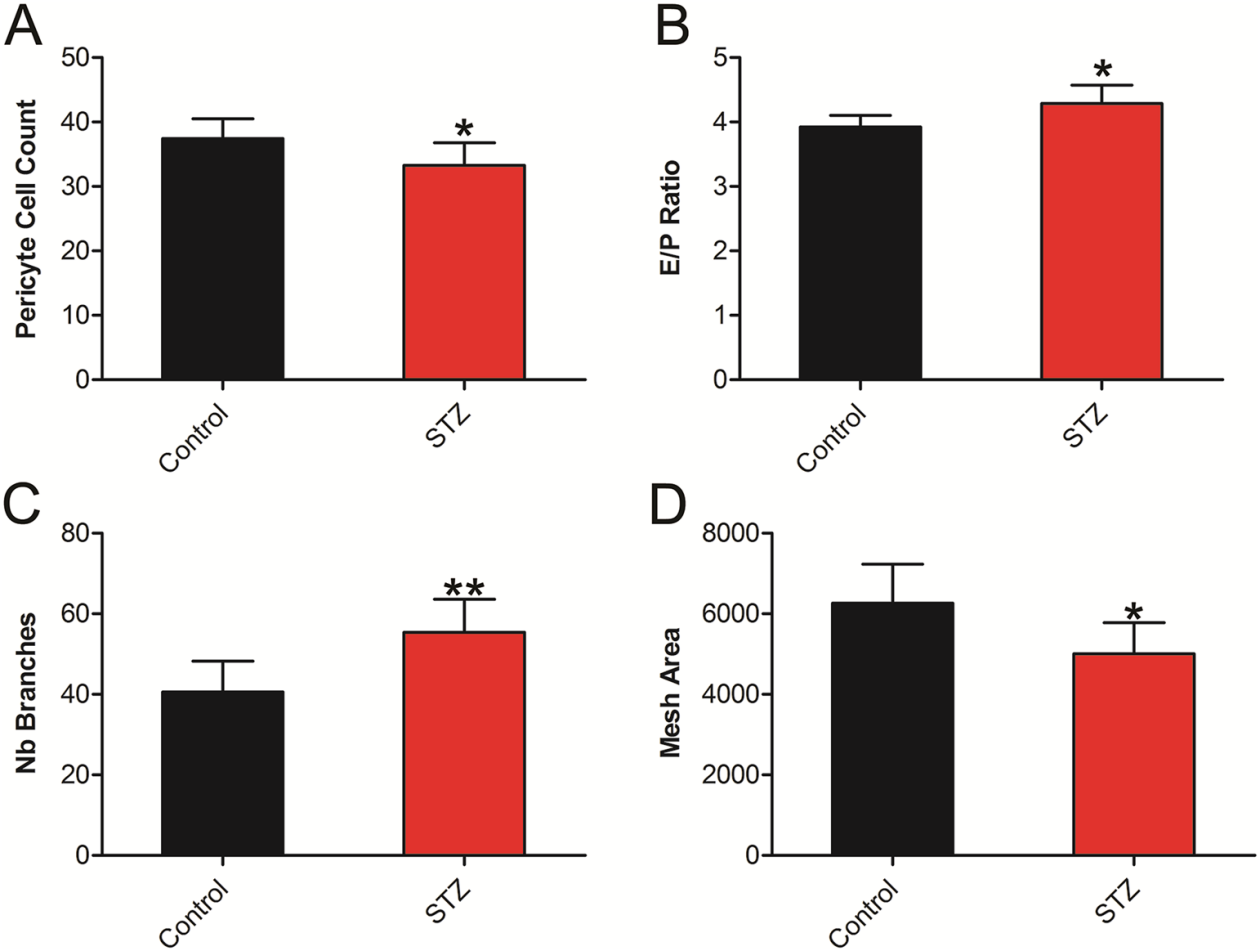
Diabetes-mediated retinal vascular changes in TSP1−/− STZ mice. (**A**) A significant decrease in pericytes cell counts was determined in TSP1−/− STZ mice compared with non-diabetic control mice (WT: 37.43 ± 3.10, STZ: 33.25 ± 3.54 (P = 0.0312). (**B**) A significant increase in E/P ratio was observed in TSP1−/− diabetic mice compared with control mice (WT: 3.92 ± 0.179, STZ: 4.29 ± 0.286 (P = 0.0130). (**C**) TSP1−/− STZ mice had a significantly greater number of branches than the control mice (WT: 40.63 ± 7.59, STZ: 55.42 ± 8.19 (P = 0.0032). (**D**) The control mice had significantly greater total mesh area than STZ mice (WT: 6266 ± 961.6, STZ 5007 ± 768.1 (P = 0.0145).

To provide further support for our findings, we similarly analyzed fundus images from non-diabetic TSP1−/− or TSP1−/− mice made diabetic by a single injection of streptozotocin. The results of noninvasive image analysis was compared with the results of trypsin digest retinal preparations from mice with 12 weeks of diabetes. Figure 5 shows diabetic mice had significantly lower pericyte counts compared with non-diabetic control mice (P = 0.0312). The endothelial/pericytes (E/P) ratio was significantly greater in diabetic mice compared with the control non-diabetic mice (P = 0.0130). The analysis of fundus images using our described method indicated a significantly greater number of branches at 12 weeks (P = 0.0032) for diabetic mice compared with non-diabetic mice. Moreover, the total mesh area was significantly lower in diabetic mice compared to control non-diabetic mice (P = 0.0145) (Fig. 5).

## Discussion

The blood vessels present in the retina are affected by many systemic diseases and could act as indicators for these pathologies. The density and architecture of vasculature are vital factors that indicate pathological conditions such as healing, trauma, and inflammation^40^. Diabetic retinopathy is one such condition where the vessels can frequently exhibit irregularities at the early stages of the disease^41^. These blood vessels can also be influenced by hypertension^42^. Retinal geometry can provide substantial insight into the body’s systemic condition where features like vessel width, neovascularization, and blood vessel occlusion are largely affected. Radial arteries tend to dilate by approximately 35% due to hypertension^43^, and its bifurcation geometry changes due to age and hypertension^44^. Angiogenesis has been closely linked with diabetes and has been extensively investigated. Diabetes doesn’t just cause vasodegeneration, but also impaires new vessel growth—angiogenesis. Thus, fundus images could provide a unique insight to these changes^45^.

There have been numerous studies attempting to automate the process of retinal blood vessel analysis but have faced challenges concerning inaccurate segmentation of the vessels and improper alignment of the images. These have resulted in a few studies describing a fully automated approach for detection and analysis of early changes in the retinal vasculature, even though there have been studies proposed for the segmentation of vasculature from the fundus images^46–52^. One study used manual alternating flicker animation to find changes in the optic nerve head^53^, whereas another study described a semi-automated method to accomplish this^54^. Other studies describe global properties of the retinal vasculature like average width or the width ratios of the vasculature and conduct a longitudinal study to detect changes in the average vessel width^55–57^. Retinal vessel tortuosity and retinal vascular fractal dimensions have also been intensely studied^58–60^. Another study describes vessel width differences from multiple frame shots of the fundus images^61^. All of these studies concentrated on different features of the retinal vasculature where one study describes a method to find fluorescein leakage in the vessels^62,63^, one studies changes in drusen^64^, and some study the methods for segmenting lesions^65–67^ and microaneurysms^63,68^. However, these methods describe global features, and are unable to pinpoint the exact location of the changes.

DR is one of the most common microvascular complication of diabetes, and there have been studies describing mechanisms for using the changes in fundus images as a mean to detect and classify DR. DR is the leading cause of new-onset blindness with 1 in every 12 person with DR losing vision in the US making it vital to find not just a robust, but an efficient method to study this disease^69,70^. Most fundus image studies related to DR investigate the pathologies like microaneurysms (MA), hemorrhages, hard exudates, neovascularization, and other irreversable microvascular abnormalities. One study proposed the method for automated detection of MA using machine learning^71^. Another study outlined the method of using deep convolutional neural networks for the recognition of DR in fundus images, which could identify DR and diabetic macular edema automatically^72^. A study done by Agurto et al.^73^ suggested a method of using a multiscale amplitude-modulation-frequency-modulation approach to differentiate between pathological pictures of the retina and normal ones. These studies particularly concentrated on irreversible abnormalities which develop with longer duration of diabetes without being able to address the underlying changes that drive these pathologies at early stages.

The majority of published studies have concentrated on fixed pathological aspects of the retinal vasculature with diabetes. The geometric features of the vasculature have their own story to tell and can be beneficial features in detecting very early stages of DR. A study was carried out to detect geometric relationships of features in the fundus images for analysis. This study also attempted to use these features to determine the severity of diabetic macular edema for early detection of DR, where they found that the exudates occurring in the macular region were more of a cause for concern than the ones far away and warranted immediate medical attention^74^. While all of the studies mentioned above have their strengths, the non-invasive detection studies described here outline a method with multiple features which is not restricted to a single or specific algorithm to do the temporal retinal analysis, nor does it only concentrate on pathological features. We have also outlined multiple features that can potentially act as biomarkers for the development and progression of DR and consequently DM noninvasively.

According to a study reported by Joshua et al.^75^, angiogenic parameters like extremities, nodes, junctions, master junction, master segments, master segment length, meshes, total mesh area, number of segments, branches, number of isolates segments, total length, total branching length, total segments length, total branches length, total isolated branches length, branching interval, total segments length /branches, mesh index, master segments length /master segments and mean mesh size could be successfully used to study the inhibition of angiogenesis in a chick embryo using an Angiogenic Analyzer software. These parameters were all used to show 50–60% inhibition of blood vessel formation in the sample where the angiogenesis inhibitor was tested. This study also successfully showed that the systematic analysis of the angiogenic index created could be used to detect vessel networks and analyze the vascular organization in the endothelial cells. Given the angioregulatory impact of diabetes, these features were chosen to determine retinal vascular changes in the mouse fundus images.

Here we demonstrate that ocular fundus images can be used to extract useful information regarding potential early temporal retinal vascular changes during diabetes. Our results indicated that the total vascular network does not decrease in diabetic mice, as indicated by the similar number of pieces and segments remaining in both diabetic and non-diabetic mice. These finding are in agreement with findings from previous studies, as significant capillary degeneration is not expected until 7–10 months following diabetes onset^24,76^. However, the decreased mesh area, mean mesh size, and number of nodes in diabetic mice suggested detectable vascular network changes in fundus images from Akita/+ mice, which appeared more disjointed. These findings are further supported with the observed increases noted in isolated segments, number of branches, and number of junctions in diabetic mice.

While the overall levels of detectable vascular network between diabetic and non-diabetic mice remained constant, our analysis detected more gaps in the diabetic vascular network compared to non-diabetic mice. The increased disjunction captured by our program aligns with current knowledge on the temporal vascular changes associated with diabetic retinopathy. Most notably, vascular leakage^77^ combined with possible pericyte loss due to engagement of inflammatory and oxidative stress mechanisms^15–17^, both of which may contribute to increased diabetic vascular disjunction initially observed. The severity of these early diabetic retinopathy markers increased over time, which was further reflected in the increased vascular disjunction over time in our fundus images. Specifically, diabetic mice have greater total number of branches and reduced branching interval compared with non-diabetic mice with longer duration of diabetes. While future studies may further elucidate specific physiological reasons underlying the observed increases in vascular disjunctions, our findings suggest fundus imaging coupled with ImageJ analysis can effectively identify vascular changes in diabetic and non-diabetic mice. The observed disjunction aligns with current publications on temporal vascular changes during diabetes.

The results obtained using TSP1−/− STZ diabetic model verified that our novel combination of the Canny Edge Detector and Angiogenesis Analyzer could successfully monitor DR in an alternative diabetes model. More importantly, our results with the STZ-diabetic mice serve as a proof of concept for physiological changes in the retina microvasculature leading to observed quantifiable differences in the fundus images. Namely, we provide evidence that the increased total number of branches and decreased total mesh area in STZ mice correlated with early pericytes loss and increases in the E/P ratio (Fig. 5). Combined with previously mentioned glucose-induced pathways, which result in retinal vascular dysfunction, there appears to be sufficient physiological basis for our observed fundus image differences in both the TSP1−/− and Akita/+ diabetic mice.

Using TSP1−/− mice, we further identified specific metrics, which were significantly different in both TSP1−/− and Akita/+ diabetic mice. Two of the metrics, which had significant differences in both diabetic models were the number of branches and the total mesh area. Although it seems likely that an increased duration of diabetes would lead to greater differences between the diabetic and non-diabetic mice, the number of branches and total mesh area metrics provide an early opportunity to identify, quantify, and track diabetes progression. Combined with the known physiological pathways, which help explain the observed differences, the number of branches and total mesh area appear to be promising metrics for early computerized quantification of diabetic retinopathy.

Fundus analysis via the Canny Edge Detector and Angiogenesis Analyzer plug-in in ImageJ could effectively identify and track other ocular diseases, which follow similar temporal vascular changes^78^. Thus far, investigators^79–81^ have used other imaging modalities, but most of these tools are not readily available to researchers and are technically challenging. The current study uses the software that was developed by the NIH and a plug-in that is freely accessible to scientists and researchers worldwide. It introduces a new application for this software with minimum human interference thus reducing the scope for human errors. Several ocular diseases where peripheral vessel leakage leads to neovascularization follow similar progression as DR by thickening of the basement membranes and loss of pericytes occurring early in their onset^82^. While our results directly support the analysis of fundus images using the Canny Edge Detector and Angiogenesis Analyzer for noninvasive monitoring of diabetes progression, the combination of these plugins may further prove to be a precise, noninvasive method for tracking other ocular, as well as non-ocular systemic diseases. Although the current investigation is performed in mice, as a proof of concept to justify the start of human studies by developing related methods and determining whether one can distinguish a patient with diabetic retinopathy form non-diabetic humans at very early stages. To our knowledge, no one has used such a methodology to temporally examine diabetes changes in retinal vasculature at very early stages of the disease. One of the limitations of the current study is that it uses only 2D fundus images and skeletonizes the blood vessels for extraction of the features. Unfortunately, this process results in the loss of some important features including the color of area of interest. Future studies will focus on eliminating the loss of these features to generate more accurate results.

The retinal vasculature appears very important in the early detection, monitoring, and prevention of many diseases including diabetes. To improve the precision and accuracy in different clinical assessments and to help with expert diagnosis of retinal vasculature integrity, manual procedures are being replaced by computer-aided diagnosis (CAD) systems^83,84^. CAD systems and other independent decision making systems aid in the interpretation of medical images, which is very effective in helping medical professionals with earlier diagnosis and treatment of patients^85^. In the case of diabetes, early detection is an important step towards preserving the health of individuals with diabetes. Premature heart disease, stroke, blindness, limb amputations, and kidney failure are all examples of serious risks associated with undiagnosed and poorly managed diabetes. The risk of these complications could be vastly reduced by early detection and development of intervention plans. The precise ability to identify diabetes mediated vascular changes in fundus images may allow the identification of underlying mechanisms. This knowledge will provide novel target for therapeutic development, and will be further advanced by advances in machine learning and artificial inelegance for accurate identification of changes^86,87^. The current study aimed to determine the factors, which are different, and how to effectively extract them using the proposed tool. Although outside the scope of the present pilot study, future studies should determine retinal differential gene expression at desired time points in an attempt to link the specific changes to activation or inactivation specific pathways. The results of such studies could aid in better delineating the origins of the retinal vascular changes with a longer duration of diabetes.

## Conclusions

Within the limitations of this study being conducted in mouse models, the studies presented here demonstrate that retinal fundus images can be utilized for the detection of early changes in the retinal vasculature integrity and function during diabetes in Akita/+ and diabetic TSP1−/− mouse models. Further studies need to be conducted to verify the efficacy of the described methods in humans by conducting clinical trials. If successful, this imaging modality coupled with the simple analysis outlined here could be effective in the assessment of therapeutic interventions. The current study was a pilot study in mouse diabetes models indicating that the ImageJ plug-in (Angiogenesis) can extract many features form 2D fundus images which can be used for quantitative analyses. Future studies could potentially apply these features to other ocular or systemic diseases. The detailed analysis of molecular and cellular mechanisms responsible for these changes could provide appropriate targets for the development of new therapeutic interventions and early detection and treatment of diabetes adverse effects on retinal vasculature as well as the vasculatures of other organs including brain and kidney.

## Supplementary information

Supplementary file1
