# Supplementary material for: Noninvasive temporal detection of early retinal vascular changes during diabetes
Source: Sci Rep. 2020 Oct 15;10:17370. doi: 10.1038/s41598-020-73486-2 (PMC7567079; doi:10.1038/s41598-020-73486-2)
Supplement: Supplementary file 1 — Supplementary file1 [file 41598_2020_73486_MOESM1_ESM.pdf]

Noninvasive temporal detection of early retinal vascular changes during diabetes

Mohammad Ali Saghiri[1, 2*], Andrew Suscha[3], Shoujian Wang[3], Ali Mohammad Saghiri[4], Christine M. Sorenson[5], Nader Sheibani[3,6]

Supplemental Material 1: Steps for Canny Edge Detector and Angiogenesis Analyzer Quantification

Video link: https://drive.google.com/file/d/1XxgOKKPF-usF_ihSeHTOKR9P2hkeOjBu/view?usp=sharing

Stage 1: Preparation and Application of Canny Edge Detection.

1. Open fundus image in ImageJ
2. Go to Adjust > Brightness/Contrast
    a. Move the minimum and maximum bars such that the minimum is directly at the lower limit of the display range (left-most portion of the curve), while the maximum bar is at the upper limit of the display range (right-most portion of the curve).
    b. Select Apply
3. Go to Adjust > Color Balance
    a. Move the minimum and maximum bars such that the minimum is directly at the lower limit of the display range (left-most portion of the curve), while the maximum bar is at the upper limit of the display range (right-most portion of the curve).
    b. Select Apply
4. Return to Adjust > Brightness/Contrast
    a. Move the minimum and maximum bars such that the minimum is directly at the lower limit of the display range (left-most portion of the curve), while the maximum bar is at the upper limit of the display range (right-most portion of the curve).
    b. Select Apply
5. Select Plugins > Canny Edge Detector
    a. Set the Gaussian radius, low threshold, and high threshold such that an appropriate amount of vasculature is highlighted. These metrics may need to be adjusted depending on fundus image quality and exposure.
        i. For our purposes, Gaussian radius ~2.1, low threshold ~1.1, and high threshold ~0.6 was sufficient for many images.
    b. Make sure "Normalize Contrast" is not selected
    c. Select "OK"
    d. View the output image. If either too much vasculature or not enough vasculature is selected, undo the Canny Edge Detector and adjust Canny Edge Detector constraints as necessary.

Stage 2: Angiogenesis Analyzer

1. Select Image > Color > RGB Color
2. Select Angiogenesis Analyzer > Analyze HUVEC Phase Contrast
    a. This step may take up to 2 or 3 minutes
3. Review the output of the angiogenesis analyzer for accuracy
4. Save output image using File > Save As
5. Go to Measurement Table Manager Menu > Save a Stat Response Table
    a. Angiogenesis Analysis output metrics will be saved in this table
